# Folate intake and depressive symptoms in Japanese workers considering SES and job stress factors: J-HOPE study

**DOI:** 10.1186/1471-244X-12-33

**Published:** 2012-04-20

**Authors:** Koichi Miyaki, Yixuan Song, Nay Chi Htun, Akizumi Tsutsumi, Hideki Hashimoto, Norito Kawakami, Masaya Takahashi, Akihito Shimazu, Akiomi Inoue, Sumiko Kurioka, Takuro Shimbo

**Affiliations:** 1 Division of Clinical Epidemiology, Department of Clinical Research and Informatics, National Center for Global Health and Medicine, Toyama 1-21-1, Shinjuku-ku, Tokyo, Japan; 2 Department of Molecular Epidemiology, Medical Research Institute, Tokyo Medical and Dental University, Tokyo, Japan; 3 Department of public Health, Kitasato University School of Medecine, Sagamihara, Kanagawa, Japan; 4 Department of Health Economics and Epidemiology Research, School of Public Health, University of Tokyo, Tokyo, Japan; 5 Department of Mental Health, Tokyo University Graduate School of Medicine, Tokyo, Japan; 6 National Institute of Occupational Safety and Health, Kawasaki, Kanagawa, Japan; 7 Department of Mental Health, Institute of Industrial Ecological Sciences, University of Occupational and Environmental Health, Kitakyushu, Fukuoka, Japan; 8 Occupational Health Training Center, University of Occupational and Environmental Health, Kitakyushu, Fukuoka, Japan

## Abstract

**Background:**

Recently socioeconomic status (SES) and job stress index received more attention to affect mental health. Folate intake has been implicated to have negative association with depression. However, few studies were published for the evidence association together with the consideration of SES and job stress factors. The current study is a part of the Japanese study of Health, Occupation and Psychosocial factors related Equity (J-HOPE study) that focused on the association of social stratification and health and our objective was to clarify the association between folate intake and depressive symptoms in Japanese general workers.

**Methods:**

Subjects were 2266 workers in a Japanese nationwide company. SES and job stress factors were assessed by self-administered questionnaire. Folate intake was estimated by a validated, brief, self-administered diet history questionnaire. Depressive symptoms were measured by Kessler’s K6 questionnaire. “Individuals with depressive symptoms” was defined as K6≧9 (in K6 score of 0–24 scoring system). Multiple logistic regression and linear regression model were used to evaluate the association between folate and depressive symptoms.

**Results:**

Several SES factors (proportion of management positions, years of continuous employment, and annual household income) and folate intake were found to be significantly lower in the subjects with depressive symptom (SES factors: *p* < 0.001; folate intake: *P* = 0.001). There was an inverse, independent linear association between K6 score and folate intake after adjusting for age, sex, job stress scores (job strains, worksite supports), and SES factors (*p* = 0.010). The impact of folate intake on the prevalence of depressive symptom by a multiple logistic model was (ORs[95% CI]: 0.813 [0.664-0.994]; *P* =0.044).

**Conclusions:**

Our cross-sectional study suggested an inverse, independent relation of energy-adjusted folate intake with depression score and prevalence of depressive symptoms in Japanese workers, together with the consideration of SES and job stress factors.

## Background

Depression is the most common psychiatric condition and regarded as a major cause of disability worldwide. In Japan, the life time prevalence of major depression has been estimated at 3-7% [1]. The presence of depressive symptoms considerably increases the risk of major depression. Among many putative risk factors, folate deficiency has been recognized since the 1960s as an important contributor to the depression [2,3]. A lot of studies examining the risk of depression in the presence of low folate were performed [4-8], including in Japanese subjects. In 2006, Gilbody reported that there is accumulating evidence that low folate status is associated with depression by meta-analysis [9]. Contrast to that in these observational studies, the effects of folate supplementation on depressive symptomatology obtained from several intervention studies were inconsistent, Taylor et al. performed a systematic review and meta-analysis then suggested that folate may have a potential role as a supplement to other treatment for depression [10], although another study failed to provide evidence for the potentiation of antidepressant medication by folate + vitamin B_12_ supplementation [11].

Another risk factor of depression is low socioeconomic status (SES) [12,13]. Low-SES groups are often exposed to an accumulation of life event stressors and chronic problems related to their low-SES state, such as poor education, poor labor circumstances and unemployment, financial strain, inadequate housing, or neighbor violence. In 2003, V. Lorant reported socioeconomic inequality in depression by meta-analysis [14]. In their report, 51 studies reported an odds ratio greater than 1, of which 35 were statistically significant.

Workplace mental health has also garnered increasing attention over the past decade because depressive disorders are highly prevalent in the workplace and have an enormously negative impact on performance, productivity, absenteeism and disability costs. Several studies have found significant associations between sources of perceived stress and major depression onset [15-17], suggesting that work stress is an independent risk factor for the development of depression. The Job Demand-Control -Support (JDCS) model has dominant research on occupational stress in which three major components are used to describe workplace qualities: demands, control, and support [18]. Sanne et al. showed that high demands, low control and low support individually, but particularly combined, are risk factors for anxiety and depression [19].

Folate has been implicated to have negative association with depression, but to our knowledge, the contribution of the SES or job stress factors is still unclear. Recently, the Japanese study of Health, Occupation and Psychosocial factors related Equity (J-HOPE study) was performed to develop and expand research to elucidate mechanisms underlying the social disparity in health and establishment of measures to control over it. The current study is a part of the J-HOPE, and our objective of this study was to clarify the association between folate intake and depressive symptoms in Japanese general workers by using large-scale samples, together with the consideration of SES and job stress factors.

## Methods

### Subjects

The present cross-sectional study was based on a baseline survey of our occupational cohort study on social class and health, supported by a grant from the Ministry of Education, Culture, Sports, Science and Technology, Japan. Employee of a Japanese major manufacturing company (Headquarter is in Kyoto and the other major 11 offices were spread all over the Japan) were recruited. All workers were invited to participate, and 2266 agreed (aged from 21–65 years; response rate 90.1%). 241 of them are women and account for 10.6%. The protocol and explanation documents of our study were approved by the ethics committee of University of Tokyo School of Medicine, and written informed consent was obtained from each subject.

### Measurements

Height, weight, systolic and diastolic blood pressures, fasting blood glucose level, serum lipid levels (total cholesterol, triglyceride, high density lipoprotein [HDL] cholesterol), aspartate aminotransferase (AST), alanine aminotransferase (ALT), gamma-glutamyl transpeptidase (gamma-GTP), blood urea nitrogen (BUN), serum creatinine (Cr), and serum uric acid (UA) levels were measured in health checkups in all subjects.

Depressive symptom was assessed by using the Kessler 6 (K6) scale [20]. K6 scale is a 6-question scale to quantify the non-specific psychological distress and now is used in clinical populations to identify those people who have a serious mental illness. It comes in a self-administered version and an interviewer-administered version [21] and is ideal for monitoring prevalence of depressive disorders on a large scale. In the current study, the self-administered version was used. The Japanese version of K6 scale was developed by using the standard back-translation method and included in the World Mental Health Survey Japan (WMH-J) [22]. The WMH-J used the WMH Survey Initiative version of the Composite International Diagnostic Interview (CIDI) to assess the 30-day Diagnostic and Statistical Manual of Mental Disorders--Fourth Edition (DSM-IV). This version demonstrated screening performances equivalent to that of the original English version. There are two scoring systems for K6 based on responses of “1-5” or “0-4”, resulting in score ranges of 0–24 or 6–30, respectively. In the current study we used the 24-scoring system. A cut-offs of ≥9 for the K6 for identifying subjects at high risk of depressive symptom was suggested according to a validation report, in which the sensitivity and specificity were estimated at 77.8 and 86.4 in Japanese population [23].

Job strain score is defined as a ratio of job demands to job control followed by multiplied by 2 in order to adjust for the difference in scoring ranges between the job demand scale (12–48) and the job control scale (24–96) [24]. Worksite support score is calculated by adding the scores of supervisor support and colleague support. Job demands and control, supervisor support and colleague support were obtained from a self-administered questionnaire. Years of education, annual household income, and the number of household were assessed by the same self-administered questionnaire as above.

### Dietary intake

Dietary habits during the preceding month were assessed with a validated, brief, self-administered diet history questionnaire (BDHQ) [25].

Responses to the BDHQ were checked for completeness and, where necessary, clarified by direct questioning of the subject. The BDHQ is a 4-page structured questionnaire that enquires about the consumption frequency of a total of 56 food and beverage items, with specified serving sizes described in terms of the natural portion or the standard weight and volume measurement of servings commonly consumed in general Japanese populations. The BDHQ was developed based on a comprehensive (16-page) version of a validated self-administered diet history questionnaire [26-28]. The BDHQ includes the main food sources for the Japanese with regard to folate (vegetables [nine items: lettuces; tomatoes; dark-green leafy vegetables; cabbages; carrots and pumpkins; radishes and turnips; onions, burdocks, and lotus roots; mushrooms; and seaweeds] and green tea [one item]). The validation of the BDHQ was performed by using 16-d weighed dietary records as the gold standard [25], and Pearson correlation coefficients for folate intake in 92 Japanese men and 92 Japanese women aged from 31 to 76 years were 0.50 and 0.62, respectively. Adjusted folate intake was calculated as daily folate intake divided by daily total calories (per 1000Kcal).

### Statistics

Student’s *T* test or Mann-Whitney’s *U* test was applied to compare variables between groups after Levine’s test for equality of variance. Difference in more than 2 groups was assessed using Turkey’s post hoc test of analysis of variance (ANOVA). Odds ratios (ORs) and 95% confidence intervals (95% CIs) were calculated using logistic regression analysis. All statistical analyses were performed using SPSS for Windows version 19.0 J (IBM Corporation, New York, USA). Statistical significance was accepted for a two-tailed p-value of < 0.05.

## Results

In Table 1, the basic characteristics, SES factors and job stressor scores of the study subjects are shown. The mean (± standard deviation, SD) age and body mass index (BMI) of the total subjects (n = 2266) were 43.5 ± 9.8 years and 23.1 ± 3.3 kg/m^2^, respectively, which are typical for middle-aged Japanese. 495 subjects (21.8%) had a K6 score more than 9 and they were defined as with depressive symptom. The incidences in male and female subjects were 22.2% and 19.0%, respectively. Comparison of subjects with and without depressive symptom revealed that the subjects with depressive symptom were significantly younger than those without (41.7 ± 9.3 years vs. 44.0 ± 9.8 years, *P* < 0.001). There were no significant differences in clinical characteristics including BMI, blood pressure, serum lipid profiles and fasting plasma glucose between these two groups.

**Table 1:** Comparison for clinical characteristics, job stress, socioeconomic status between subjects with or without depressive mood

	**Total subjects (n = 2266)**	**Subjects without depressive mood (n = 1771)**	**Subjects with depressive mood (n = 495)**	**P value**
Age (year)	43.5 ± 9.8	44.0 ± 9.8	41.7 ± 9.3	<0.001**
Proportion of women (%)	10.6	11.0	9.3	0.273
Body mass index (kg/m^2^)	23.1 ± 3.3	23.0 ± 3.2	23.3 ± 3.5	0.209
Baseline characteristics				
Systolic blood pressure (mmHg)	123.4 ± 16.1	123.7 ± 16.1	122.2 ± 16.0	0.081
Diastolic blood pressure (mmHg)	77.1 ± 12.0	77.1 ± 12.0	77.0 ± 11.9	0.760
Total cholesterol (mg/dL)	200.0 ± 35.1	200.2 ± 35.2	199.1 ± 34.5	0.582
Triglyceride (mg/dL)	125.8 ± 180.0	125.4 ± 195.3	127.4 ± 104.5	0.840
HDL cholesterol (mg/dL)	61.8 ± 16.5	62.0 ± 16.3	61.2 ± 17.4	0.424
Fasting plasma glucose (mg/dL)	95.0 ± 23.2	95.1 ± 21.7	94.9 ± 28.0	0.933
AST (IU/L)	23.7 ± 18.3	23.6 ± 19.7	24.0 ± 11.8	0.752
ALT (IU/L)	25.8 ± 18.1	25.5 ± 18.1	26.7 ± 18.0	0.245
γ-GTP (IU/L)	47.4 ± 56.8	47.1 ± 55.9	48.5 ± 60.2	0.676
SES factors				
Years of education (year)	14.5 ± 2.5	14.6 ± 2.5	14.4 ± 2.5	0.320
Proportion of management position (%)	22.7	24.4	16.6	<0.001**
Years of continuous employment (year)	20.6 ± 11.6	21.1 ± 11.7	19.2 ± 11.1	0.001**
Working hours (hour/week)	46.6 ± 7.2	46.5 ± 7.1	47.0 ± 7.6	0.185
Annual household income (ten thousands yen/year)	704.4 ± 297.5	717.9 ± 303.3	656.3 ± 270.7	<0.001**
Adjusted annual household income (ten thousands yen/year)	443.0 ± 188.5	446.1 ± 188.7	431.8 ± 187.7	0.134
Number of families (n)	2.9 ± 1.4	2.9 ± 1.4	2.8 ± 1.5	0.018*
Job stress scores				
Job Demands	4.7 ± 2.5	4.5 ± 2.4	5.5 ± 2.6	<0.001**
Job Control	3.0 ± 1.4	3.0 ± 1.4	2.7 ± 1.4	<0.001**
Job Strain	4.0 ± 3.4	3.7 ± 3.1	5.2 ± 4.0	<0.001**
Worksite Support	11.8 ± 1.8	11.9 ± 1.6	11.3 ± 2.0	<0.001**
K6 Score	5.1 ± 4.6	3.2 ± 2.6	12.2 ± 3.3	<0.001**
Daily folate intake (μg/day)	295.6 ± 127.3	297.6 ± 125.9	288.5 ± 131.9	0.162
Energy-adjusted folate intake (μg/1000 kcal·day)	163.0 ± 56.9	165.0 ± 57.4	155.8 ± 54.7	0.001**

Values are shown as mean ± standard deviation or percentage. Subjects with or without depressive mood were compared. Depressive symptom is defined as K6 score ≥9 in a 24-scoring system.

Job strain ratio is defined as a ratio of job demands to job control followed by multiplied by 2. Worksite support score is calculated by adding the scores of supervisor support and colleague support.

For continuous variables we used Student’s *T* test or Mann–Whitney *U* test. As to the categorized variables, we used *χ*2 test.

*P <0.05; **P < 0.01.

As to SES factors, proportion of management positions, years of continuous employment, and unadjusted annual household income were found to be significantly lower in the subjects with depressive symptom. Also the daily folate intake levels adjusted by total energy intake were significantly different between two groups (155.8 ± 54.7 vs 165.0 ± 57.4 μg/1000 kcal·day, *P* = 0.001), although the unadjusted folate intake were not different (*P* = 0.162).

For job stressor scores, notable differences were found between subjects with or without depressive symptom in job demands, control, strain and worksite supports. All of the P values were less than 0.001.

The relationship between K6 score - the measurement of depressive symptom, and dietary folate intake level, was further assessed by linear regression model. The association of K6 score with folate intake as a continuous variable is shown in Table 2. When the analysis was conducted by using single factor, age, job strain, worksite support, proportion of management position, annual household income and energy-adjusted folate intake correlated to K6 scores, suggesting the real association with depression of each factor (For folate intake, β = −0.077, *P* <0.001, other data are not shown). If the adjusted factors include age and sex only, the relation remains significant with a P value for trend as 0.002 (β = −0.067). There was an inverse linear association between K6 score and folate intake, P value was 0.010 after adjusting for age, sex, job stress score (job strain and worksite support) and SES factors (years of education, proportion of management position and annual household income). In addition, age and both of the stressor scores were also associated with K6 score (P <0.001). In this study, neither of the SES factors influenced on K6 score. Even if the effects of confounding factors such as SES factors and job stress were considered, our results provided the evidence for the robust correlation between folate intake and depression.

**Table 2:** Association of energy-adjusted folate intake with K6 score by linear regression models

**Energy-adjusted folate intake**	**Standard β**	**P value**
Model 1	−0.077	<0.001**
Model 2	−0.067	0.002**
Model 3	−0.054	0.010*

P values (β coefficients) showing the significance for linear regression analysis are present. P for model <0.001. *P <0.05; **P < 0.01.

In Model 1, the unadjusted P value for the association between K6 score and energy-adjusted folate intake is shown. In Model 2, P value is adjusted for age and sex only. In model 3, adjust factors include age, sex, job stress scores and SES factors.

**Figure 1:**
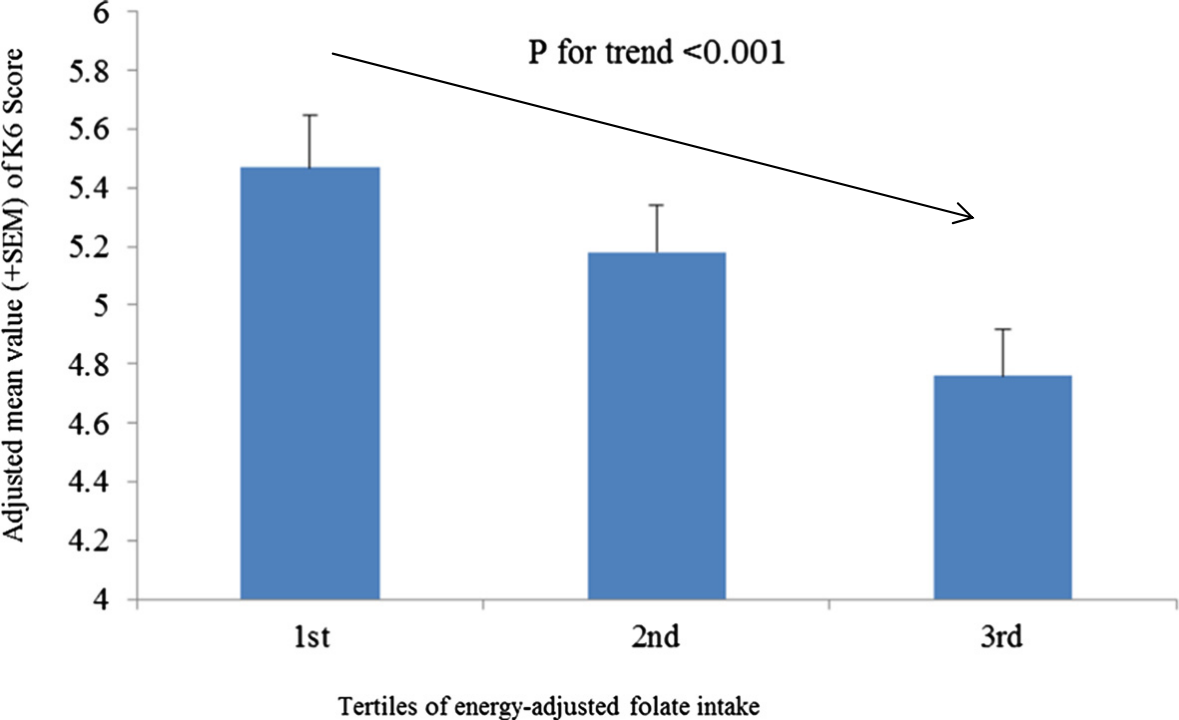
**The comparison of mean values (+SEM) of adjusted K6 score between different folate intake subgroups.** K6 score is adjusted for age, sex, job strain, worksite support, years of education, proportion of management position and annual household income.

When the subjects were classified into tertiles according to energy-adjusted folate intake level, mean K6 score (±standard error of the mean [SEM]) of each subgroup adjusted for age, sex, job strain, worksite support, years of education, proportion of management position and annual household income was calculated and compared. The adjusted mean values were 5.5 ± 0.2, 5.2 ± 0.2 and 4.8 ± 0.2, respectively. P value for the trend was less than 0.001, indicating a significant linear reduction of K6 score with the increasing of folate intake (Figure 1).

We also considered the gender difference on this association. By the separate linear regression analysis performed in two genders, the association between energy-adjusted folate intake and K6 score remained in male subjects (β = −0.050, *P* = 0.021), but disappeared in females (β = −0.054, *P* = 0.412).

Multiple logistic regression was also performed to examine whether the prevalence of depressive symptom relates to folate intake level. According to the result shown in Table 3, if the folate intake increases 100 μg per 1,000 kcal in one day, the risk of becoming depressive reduced approximately 19% (95% CI: 0.664-0.994; *P* = 0.044). Meanwhile age and worksite support also decreased the risk of depressive symptom, however, high job strain did oppositely.

**Table 3:** The multiple logistic regression analysis of energy-adjusted folate intake as to with or without depressive mood

	**Odds Ratio [95% CI]**	**P value**
Age (year)	0.971 [0.958-0.985]	<0.001**
Sex (male = 1, female = 2)	0.821 [0.570-1.182]	0.288
Job stress scores		
Job Strain	1.096 [1.065-1.128]	<0.001**
Worksite Support	0.837 [0.786-0.891]	<0.001**
SES factors		
Years of education (year)	0.985 [0.938-1.033]	0.528
Management position (others = 0, manager = 1)	0.916 [0.665-1.261]	0.590
Annual household income (million yen/year)	0.980 [0.937-1.025]	0.382
Energy-adjusted folate intake (100 μg/1000 kcal·day)	0.813 [0.664-0.994]	0.044*
Constant	13.432	<0.001**

Depressive symptom is defined as K6 score ≥9 in a 24-scoring system. CI indicates confidence interval. P for model <0.001.

*P < 0.05; **P < 0.01.

## Discussion

In the current study, we confirmed an inverse, independent linear relation of K6 score with the energy-adjusted folate intake level in Japanese workers. These results are in agreement with previous study of Japanese [5,29]. It is worthy of note that comparing with the small sample size of their studies (517 in Ref. 5 and 530 in Ref. 29), our study was a large-scale investigation with more than 2,200 participants, providing more convincing evidence for this association. Furthermore, we took various job stress scores into account as potential relative factors at the first time. When the job stress scores were not added and the analysis was adjusted for age, sex and SES factors only, the folate intake was also associated with K6 score by linear regression (standard β was −0.059; P value for trend was 0.006) and prevalence of depressive symptom by logistic regression (ORs [95% CI]: 0.813 [0.671-0.985]; P value was 0.035). After further adjusted for the job stress scores, the relations of folate intake to K6 score and prevalence of depressive symptom remained (P values are 0.010 and 0.044, respectively), and the stress factors also showed association with depressive symptom, providing inspiration to understand thoroughly the relationships among depression and relative factors.

The results that the association between folate intake and depressive mood was found in men only, were consistent with that of Murakami et al. [5] and Nanri et al. [29], who found that higher folate intake was associated with lower prevalence of depressive symptoms in Japanese men but not in women, although in another study the association was found only in USA women [7]. The possible explanation for the gender difference is the relatively sufficient folate intake in women. In the present study, mean (±SD) values of adjusted intake of men and women were 159.4 ± 54.1 and 192.9 ± 69.9 μg/1000 kcal·day, respectively, therefore the higher intakes of female subjects maybe mask the effect of folate intake on depression. However, because of the relative small size of female subjects in our study and the importance of folate intake in pregnant women, whether the dietary folate intake affect mental health should be further investigated in larger scale and well-designed studies.

The mechanism of folate intake relate to depressive symptom was thought to be that a decreased folate intake results in an accumulation of homocysteine and then elevated homocysteine levels, which cause vascular disease of the brain, and/or transmitter alterations, could cause depression [30]. Higher total plasma homocysteine concentrations were associated with older age, male gender [31] and dietary deficiency of folate, vitamins B12 and B6 [32]. It was described that the lesions of atherosclerosis could be induced by administering homocysteic acid by animal experiment [33]. Early studies suggested a relationship between depression and cerebrovascular and cardiovascular disease [34], and deficiency of folate and elevated homocysteine levels was considered to increase the risk for vascular disease of the brain [35-37]. However, more recent evidences strongly supported a different role of folate. Folate plays a role in the 1-carbon cycle, and eventually produces S-adenosyl-_L_-methionine (SAMe), which is an important methyl donor and thought to be involved in the synthesis of three key neurotransmitters in the brain-dopamine, serotonin and norepinephrine [38]. Homocysteine is a critical branch point metabolite that can influence cellular levels of SAMe and S-adenosylhomocysteine (SAH), which regulate the activity of methyltransferases important in posttranslational modification of proteins and synthesis of nucleic acids, phospholipids, and neurotransmitters [39]. Thus, low folate intakes, which result in elevated homocysteine levels, lead to a deficiency of these neurotransmitters and then cause depression. The homocysteine hypothesis of depression reviewed by Folstein et al. [30] centering around hyperhomocysteinemia leading to an increased risk of stroke, heart disease, neurotransmitter imbalances and thereby depression was further extended by a brief comment in the American Journal of Psychiatry in 2007 [40]. The authors herein stated that homocysteine is metabolized to SAMe, then influence DNA methylation. Secondly, homocysteine itself was shown to affect global and gene promoter DNA methylation, and the administration of acute homocysteine usually leads to demethylation of promoter DNA with a subsequent increase in gene expression [41]. A further study supported the hyperhomocysteine hypothesis of depression, reporting significantly higher homocysteine levels in patients with moderate depressive symptoms and eating disorder diagnoses [42]. Our observations epidemiologically confirmed with this hypothesis.

Some limitations of the present study are worth mentioning. Firstly, the cross-sectional nature of the study does not permit the assessment of causality owing to the uncertain temporality of the association. Depressed persons may eat less or more and the intakes of nutrition are influenced by the total amount of intake. In order to remove this effect, we used the total energy adjusted folate intake level (μg/1000 kcal per day). Our data showed energy adjusted low folate intake was significantly related to depressive symptoms, and the probability of the reverse causation seems slim. Secondly, our subjects were workers in one large company and not a random sample of Japanese workers, and thus the results may not apply to the general Japanese population. However, the workers were recruited from 12 offices all over the Japan (From Hokkaido to Kyushu). So the geographical deviation was reasonably diluted, but it is noteworthy fact that our result is the result of a large company, not a small company. Finally, dietary data were obtained from a self-administered semi-quantitative dietary assessment questionnaire [25]. Because the actual dietary habits were not observed, the results should be interpreted with caution, although the validity of this questionnaire appears reasonable [26-28].

## Conclusions

In conclusion, low folate intake was independently related to higher depression scores and increased prevalence of depressive symptoms in Japanese workers, adjusting for job strains, supports and various SES factors. Further prospective or intervention studies are warranted to determine this association as well as the roles of SES and stress effect on mental health outcomes.

## Abbreviations

SES, Socioeconomic status; JDCS, Job Demand-Control –Support; HDL, High density lipoprotein; AST, Aspartate aminotransferase; ALT, Alanine aminotransferase; gamma-GTP, gamma-glutamyl transpeptidase; BUN, Blood urea nitrogen; Cr, Serum creatinine; UA, Serum uric acid; K6 scale, Kessler 6 scale; BDHQ, Brief-type self-administered diet history questionnaire; ANOVA, Analysis of variance; ORs, Odds ratios; CIs, Confidence intervals; SD, Standard deviation; BMI, Body mass index; SEM, Standard error of the mean; SAM, S-adenosyl-L-methionine; SAH, S-adenosylhomocysteine..

## Competing interests

The authors declare that they have no competing interests.

## Authors’ contributions

KM conceived the study, analyzed the data, and drafted the manuscript. YS and NCH coordinated data collection, participated in the analysis and edited the manuscript. AT, HH, NK and TS participated in the maintenance of the cohort and data collection, provided critical advices. MT, AS, AI and SK coordinated the maintenance of the cohort and data collection. Finally, all authors read and approved the final manuscript.

## Pre-publication history

The pre-publication history for this paper can be accessed here:

http://www.biomedcentral.com/1471-244X/12/33/prepub
